# Incorporation of Peptides Targeting EGFR and FGFR1 into the Adenoviral Fiber Knob Domain and Their Evaluation as Targeted Cancer Therapies

**DOI:** 10.1089/hum.2015.015

**Published:** 2015-04-27

**Authors:** Hanni Uusi-Kerttula, Mateusz Legut, James Davies, Rachel Jones, Emma Hudson, Louise Hanna, Richard J. Stanton, John D. Chester, Alan L. Parker

**Affiliations:** ^1^ Institute of Cancer and Genetics, Cardiff University, Cardiff CF14 4XN, United Kingdom.; ^2^ Institute of Infection and Immunity, School of Medicine, Cardiff University, Cardiff CF14 4XN, United Kingdom.; ^3^ Velindre Cancer Centre, Cardiff CF14 2TL, United Kingdom.

## Abstract

Oncolytic virotherapies based on adenovirus 5 (Ad5) hold promise as adjunctive cancer therapies; however, their efficacy when delivered systemically is hampered by poor target cell specificity and preexisting anti-Ad5 immunity. Ovarian cancer represents a promising target for virotherapy, since the virus can be delivered locally into the peritoneal cavity. Both epidermal growth factor receptor (EGFR) and fibroblast growth factor receptor 1 (FGFR1) are overexpressed in the majority of human tumors, including ovarian cancer. To generate adenoviral vectors with improved tumor specificity, we generated a panel of Ad5 vectors with altered tropism for EGFR and FGFR, rather than the natural Ad5 receptor, hCAR. We have included mutations within AB loop of the viral fiber knob (KO1 mutation) to preclude interaction with hCAR, combined with insertions in the HI loop to incorporate peptides that bind either EGFR (peptide YHWYGYTPQNVI, GE11) or FGFR1 (peptides MQLPLAT, M*, and LSPPRYP, LS). Viruses were produced to high titers, and the integrity of the fiber protein was validated by Western blotting. The KO1 mutation efficiently ablated hCAR interactions, and significantly increased transduction was observed in hCAR^low^/EGFR^high^ cell lines using Ad5.GE11, while transduction levels using Ad5.M* or Ad5.LS were not increased. In the presence of physiological concentrations of human blood clotting factor X (hFX), significantly increased levels of transduction via the hFX-mediated pathway were observed in cell lines, but not in primary tumor cells derived from epithelial ovarian cancer (EOC) ascites samples. Ad5-mediated transduction of EOC cells was completely abolished by the presence of 2.5% serum from patients, while, surprisingly, incorporation of the GE11 peptide resulted in significant evasion of neutralization in the same samples. We thus speculate that incorporation of the YHWYGYTPQNVI dodecapeptide within the fiber knob domain may provide a novel means of circumventing preexisting Ad5 immunity that warrants further investigation.

## Introduction

Ovarian cancer remains the fourth most common cancer in women in the United Kingdom, with ∼7000 women diagnosed annually with the disease, and a mortality rate of ∼4500 per annum. Patients often present with advanced disease because of the relatively nonspecific symptoms associated with the disease, even in the early stages. Despite recent therapeutic advances with the role of neoadjuvant chemotherapy, changes in chemotherapy scheduling, bevacizumab,^1^ and PARP inhibitors,^2^ the outlook for advanced ovarian cancer patients remains poor, with only small improvement in 5-year survival statistics over the last 20 years. Standard treatments remain surgery and/or chemotherapy, and despite good initial responses to chemotherapy, many tumors rapidly develop resistance and progress into aggressive, platinum-resistant forms. Therefore, there is a pressing need to establish new therapeutics to combat the disease.

Oncolytic virotherapy is a promising adjunct to conventional drug-based strategies for effective cancer therapies, and has progressed into late-phase clinical trials.^3^ An oncolytic herpes virus expressing GM-CSF has demonstrated higher response rates (26% vs. 6%) in malignant melanoma with durable clinical responses, lasting for a minimum of 6 months, in 16% of patients, compared with 2% in the control arm (GM-CSF treatment alone). These encouraging clinical data have helped to re-energize the field of oncolytic virotherapy for cancer.

Among the repertoire of viruses under investigation as oncolytics, human adenoviruses (Ad) have been widely studied clinically and are generally well-tolerated and efficient.^4^ Viruses can be grown and purified to exceptionally high titers (>10^13^ viral particles/ml), and are readily amenable to genetic modulation. Because of this versatility, they represent the most commonly deployed virus clinically, and are represented in 23% of gene therapy clinical trials. The Ad phylogenetic tree is diverse, with 57 serotypes described to date, divided into 7 species, A–G, based on receptor usage, hemagglutination patterns, serological cross reactivity, and phylogenetic sequence alignments. The species C serotype, adenovirus 5 (Ad5), is by far the most commonly used, in both research and clinical trials. However, the primary Ad5 receptor, coxsackie and adenovirus receptor (hCAR), is ubiquitously expressed in all organs of the human body and on erythrocytes.^5,6^ Physiologically, its expression pattern in organs co-localizes with tight junction marker, zona occludens ZO-1, and is thus restricted to tight junctions.^7^ Furthermore, there is increasing evidence suggesting that expression of hCAR correlates negatively with tumor progression in certain cancers.^8–10^

Targeted, tumor-specific delivery of Ad5-based virotherapy utilizing an unmodified hCAR is therefore challenging. Furthermore, since Ad5 is a common pathogen of the respiratory tract, up to 90% of the population have high titers of preexisting neutralizing antibodies (nAbs) against Ad5, resulting in rapid and efficient elimination and neutralization of Ad5-based vectors when delivered systemically.^11,12^ Such high level of preexisting immunity may significantly hamper clinical translation and efficacy of Ad5 vectors, and consequently a great deal of effort has therefore been placed on developing means to evade preexisting Ad5 immunity, either genetically^13^ or chemically, using hydrophilic polymers such as pHPMA^14^ or PEG.^15^

Ovarian cancer frequently presents as stage 3 disease, with peritoneal metastases. This disease state represents a good potential target for intraperitoneal delivery of Ad-based virotherapy. Local delivery precludes the requirement for intravenous delivery, where interactions with multiple cell types and a variety of serum proteins, including complement-related proteins and blood clotting proteins, in particular human blood clotting factor X (hFX), dictate toxicity and tropism by “bridging” the virus to heparan sulfate proteoglycans (HSPGs),^16–19^ abundant on hepatocytes. We hypothesized that combining mutations in the Ad5 genome that abrogate hCAR interactions (KO1 mutation), with insertion of peptide motifs into the viral fiber knob that are selective for tumor-associated receptors, would generate Ad vectors with improved tumor selectivity in ovarian cancer. Previous studies have identified key hCAR-interacting amino acids within the fiber AB loop at Ser408 and Pro409.^20^ Furthermore, genetic re-targeting via peptide insertion in the HI loop (after Thr541) has been shown previously to be feasible.^21^ We selected three previously described peptide agonists, selected from phage libraries, for genetic insertion into the Ad5 HI loop: a dodecapeptide YHWYGYTPQNVI (GE11) binding to epidermal growth factor receptor (EGFR)^22^ and two heptapeptides, MQLPLAT (M*)^23^ and LSPPRYP (LS),^24^ binding to fibroblast growth factor receptor 1 (FGFR1). EGFR is overexpressed in numerous tumor types, including lung, head and neck, colon, pancreas, breast, ovary, bladder, and kidney cancer (reviewed in ref.^25^). Approximately 30% of patients with both primary and recurrent ovarian serous cancers demonstrate EGFR overexpression.^26^ Similarly, the FGFR signaling pathway has been shown to be upregulated in the majority of human tumor types.^27^

Here, we report the successful production and *in vitro* characterization of a suite of six novel, potentially tumor-targetable recombinant Ad5 vectors. We evaluate their ability to transduce cancer cell lines *in vitro*, and evaluate the effect of hFX on their capacity to transduce both cell lines and primary, epithelial ovarian cancer (EOC) cells isolated from ascites of patients with ovarian cancer. Importantly, to best replicate the clinical scenario, we evaluate the capacity of neutralizing serum from ascites to neutralize Ad5 transduction of patient-derived EOC *in vitro* and outline a potential role for peptide incorporation in protecting Ad5-based vectors from nAbs.

## Materials and Methods

### Cell lines

CHO-K1 (Chinese hamster ovarian epithelial) and CHO-CAR (CHO-K1 cell line transfected to express hCAR) cells were grown in Dulbecco's modified Eagle's medium (DMEM)/F12. T24 (human transitional cell carcinoma), SKOV3 (human ovarian adenocarcinoma), and T-REx-293 (human embryonic kidney) cells were all grown in DMEM. RPMI 1640 medium was used for the culture of OVCAR3 (human ovarian adenocarcinoma) cells. All cell culture media were supplemented with 4 m*M* L-Glutamine, 100 U/ml penicillin, 100 μg/ml streptomycin, and 10% fetal calf serum (FCS; except 20% for OVCAR3). Dulbecco's phosphate buffered saline and 0.05% trypsin were used for subculture, all cells were grown at 37°C in a humidified atmosphere with 5% CO_2_, and all reagents were purchased from Gibco or Thermo Scientific (Paisley, UK).

### Flow cytometry

An amount of 2.5×10^5^ cells were incubated with primary antibodies in triplicate: mouse anti-CAR clone RcmB (1:500; Millipore, Watford, UK), mouse anti-EGFR clone H11 (1:200; Thermo Scientific), mouse anti-FGFR1 clone M19B2 (1:100; Abcam, Cambridge, UK), and normal mouse IgG control (1:200; Santa Cruz Biotechnology, Heidelberg, Germany) on ice for 1 h, followed by incubation with a secondary goat antimouse IgG Alexa Fluor647-conjugated antibody (1:500; Life Technologies, Paisley, UK) for 30 min on ice. The cells were fixed in ice-cold 2% paraformaldehyde (PFA), 2×10^4^ gated events were acquired on BD Accuri C6 (BD Biosciences) flow cytometer, and data analysis was performed using FlowJo software (Tree Star, Ashland, OR).

### Primary cell culture from ovarian ascites

Permission for the collection and cultivation of cells from ovarian ascites was granted through a Wales Cancer Bank application for biomaterials, reference WCB 14/004. All patients gave written informed consent for the use of their samples before collection. A 500 ml total volume of ovarian ascites (OAS) clinical samples were received from Velindre Cancer Centre, Cardiff, and assigned as “group 2” according to the stage of chemotherapy (1, pretreatment/chemo naïve; 2, first-line chemo; 3, relapsed disease/platinum sensitive; 4, relapsed disease/platinum resistant). Samples were given codes OAS000 and OAS001 to retain anonymity. Fluids were stored at 4°C following collection from the patient, and processed fresh within 2 hr. Primary EOC cells were separated from the fluid by centrifugation at 1000 rpm for 5 min. The ascites fluid supernatant was stored at −70°C and the cell pellet was treated twice with 2 ml of red blood cell lysis buffer (Sigma Aldrich, Gillingham, UK) according to the manufacturer's protocol. Two-thirds of the cell pellet was aliquoted and frozen in 10% dimethyl sulfoxide and 90% autologous supernatant at −70°C (passage 0) and 1/3 of the cell pellet was resuspended into 10 ml of RPMI 1640 medium supplemented with 4 m*M* Glutamax, 10% FCS, 100 U/ml penicillin, 100 μg/ml streptomycin, 2.5 μg/ml of amphotericin B, and 10% of the autologous fluid supernatant and cultured in a T25 cell culture flask. On the following day, the cells were washed with PBS to remove the nonattached contaminating cells and cell debris, and supplied with 10 ml of fresh medium. The cells were then subcultured or fed every 3–5 days. Cell morphology was monitored by visual examination under a light microscope throughout cell passages to exclude the possibility of fibroblast contamination.

### 3D modeling of the recombinant fiber proteins

The Ad5 fiber knob domains (GenPept: AP_000226.1) with peptide insertions (scramble, GE11, M*, and LS) were modeled by using SWISS-MODEL software^28–30^ (Basel, Switzerland) and edited in PyMol Molecular Graphics software (version 1.1eval, Schrödinger, LLC, NY) to assess the spatial conformation of the inserted peptides in the context of the homotrimeric knob structure. The models of the recombinant knobs are based on the wild-type Ad5 fiber knob template (Protein Data Bank ID: 1KNB).

### Generation of recombinant adenovirus genomes

The recombinant Ad5 genomes were based on luciferase (Luc)-expressing replication-deficient (ΔE1/ΔE3) Ad5 (referred to as Ad5.Luc throughout the article).^31^ DNA sequences coding for EGFR and FGFR1 binding peptides were inserted into the HI loop and a hCAR-binding ablating KO1 mutation (S408E, P409A)^32^ was introduced into the AB loop of Ad5 fiber knob. The Ad5 genome was modified by homologous recombination in *Escherichia coli* strain SW102 in a two-step process. First, an RPSL/neo selection cassette was inserted into the region of interest, and then replaced with DNA containing the appropriate sequence. Selection cassettes and targeting peptide encoding DNA fragments containing 70 bp homology arms were generated by PCR using Expand Hi-Fi PCR system (Roche Applied Science, East Sussex, UK). To generate the KO1 mutation, a 100 bp oligonucleotide (Sigma Aldrich) was used in the second recombination step (Supplementary Table S1A; Supplementary Data are available online at www.liebertpub.com/hum). To confirm correct recombination, the DNA constructs were sequenced (Supplementary Table S1B) in an ABI Prism 3130 genetic analyzer (Applied Biosystems, Warrington, UK). Cycle sequencing was performed according to manufacturer's instructions, with the exception that 100 cycles were used instead of 25. Sequencing reactions were purified using Performa DTR columns (Edge Biosystems, Gaithersburg, MD) and sequence verification was performed with CLC Main Workbench 6 software (Qiagen, Manchester, UK).

### Production and purification of Ad5 vectors

For virus generation, DNA was purified from 250 ml overnight culture (BacMax 100 kit; Macherey-Nagel, Duren, Germany) and transfected into T-REx-293 cells in T25 tissue culture flasks (Corning CellBIND, Amsterdam, The Netherlands) using Effectene (Qiagen). When cytopathic effect (CPE) was complete, cell pellets were collected and virus extracted with tetrachloroethylene (Fisher Scientific, Loughborough, UK). To generate a working stock, this virus preparation was used to infect 5× T150 confluent T-Rex-293 flasks. Cell pellets were collected when CPE was complete (3–7 days postinfection), and tetrachloroethylene extraction was performed. This crude virus was purified by two rounds of cesium chloride (CsCl) gradient ultracentrifugation and dialyzed against buffer containing 10% glycerol, 10 m*M* Tris-HCl (pH 7.8), 135 m*M* NaCl, and 1 m*M* MgCl_2_*6H_2_O to remove CsCl. Viral titers were determined using microbicinchoninic acid (BCA) assay (Pierce, Thermo Scientific) assuming that 1 μg protein equals 4×10^9^ viral particles (vp).^33^

### Western blotting to detect Ad5 fiber protein

The structural integrity of the Ad5 fiber proteins was assessed by Western blotting. An amount of 5×10^9^ vp/virus stock were run on ready-made 10% NuPAGE polyacrylamide gels (Invitrogen, Paisley, UK) by SDS-PAGE and transferred to Hybond-P nitrocellulose membrane (GE Healthcare Life Sciences, Little Chalfont, UK) by semidry blotting. Nitrocellulose membranes were treated with 5 ml of Pierce Miser antibody extender (Thermo Scientific) for 10 min and washing 7 times with distilled water. They were then blocked in 5% milk in Tris-buffered saline containing 0.05% TWEEN-20 and 0.05% Triton X-100 (TBS-T) overnight at 4°C. The membrane was incubated in primary anti-adenovirus fiber antibody 4D2 (1:2000) at 37°C for 1 hr, washed 5 times for 5 min in TBS-T, and incubated in antimouse IgG-HRP conjugate (1:2000; Insight Biotechnology Ltd., Wembley, UK) for 1 hr at room temperature. After washing a further 5 times for 5 min in TBS-T, the membrane was incubated for 5 min in SuperSignal West Pico Chemiluminescent substrate (Thermo Scientific), and analyzed on GelDoc autoChemi camera (Ultra-Violet Products Ltd., Cambridge, UK).

### Transduction (luciferase) assays

The assay was performed using the Luciferase Assay System kit, according to the manufacturer's protocol (Promega UK Ltd., Southampton, UK) with slight modifications. In brief, 24 hr before the experiment, cells were seeded on a 96-microwell plate (Nunc, Thermo Scientific) in complete growth medium at a density of 2×10^4^ cells/well in triplicate and allowed to adhere overnight at 37°C. Medium was then removed, cells were washed with 200 μl of PBS, and viruses were added at doses of 1×10^3^, 5×10^3^, and 1×10^4^ vp/cell in serum-free (serum^−^) DMEM for 3 hr, after which the medium was replaced with cell line-specific complete growth media. Forty-eight hours postinfection the cells were lysed with 1× Cell Culture Lysis Buffer (Promega UK Ltd.) and frozen at −70°C. The cells were thawed and prepared for the analysis by Luciferase Assay System so that 20 μl of cell lysate was mixed with 100 μl of luciferase assay reagent in a white 96-well microwell plate and the luciferase activity in relative light units (RLU) was then immediately measured on a multimode plate reader (FLUOstar Omega; BMG Labtech, Aylesbury, UK). Protein concentration in each well of the sample plate was determined using microBCA assay kit and bovine serum albumin (BSA) as a protein standard. Luciferase activity from each well was then normalized for total cellular protein (RLU/mg).

### Transduction assays in the presence of hFX and ovarian ascites fluid

Transduction assays in the presence of hFX were performed essentially as described for the luciferase assay above, with the exception of virus preincubation in serum^−^ medium supplemented with 10 μg/ml of hFX (Haematologic Technologies, Cambridge Bioscience, Cambridge, UK) for 3 hr. All viruses were assayed at a dose of 5×10^3^ vp/cell in quadruplicate on OVCAR3 and T24 cell lines on a 96-well plate (Nunc, Thermo Scientific). For the transduction neutralization assays on primary EOC cells (passage 5), the viruses were preincubated in serum^−^ medium supplemented with 2.5% of the OAS000 or OAS001 ascites supernatants in triplicate on a 96-well plate. Ascites supernatant that exhibited >90% inhibition of transduction was considered to be neutralizing, as described and validated previously for serum neutralization assays.^11^

### Statistical analysis

All figures were created in GraphPad Prism version 4.03 (GraphPad Software Inc., La Jolla, CA) and statistical analysis performed using GraphPad Quickcalcs *t*-test calculator. Unless otherwise stated, data show the mean+SD of *n*=3–4 (specific *n* numbers are indicated in each figure legend). Statistical significance was determined using two-tailed unpaired *t*-test. *p-Value of <0.05, **p<0.01, ***p<0.001, ns=not statistically significant, *p*>0.05.

## Results

### Generation of recombinant adenoviruses

**FIG. 1.**
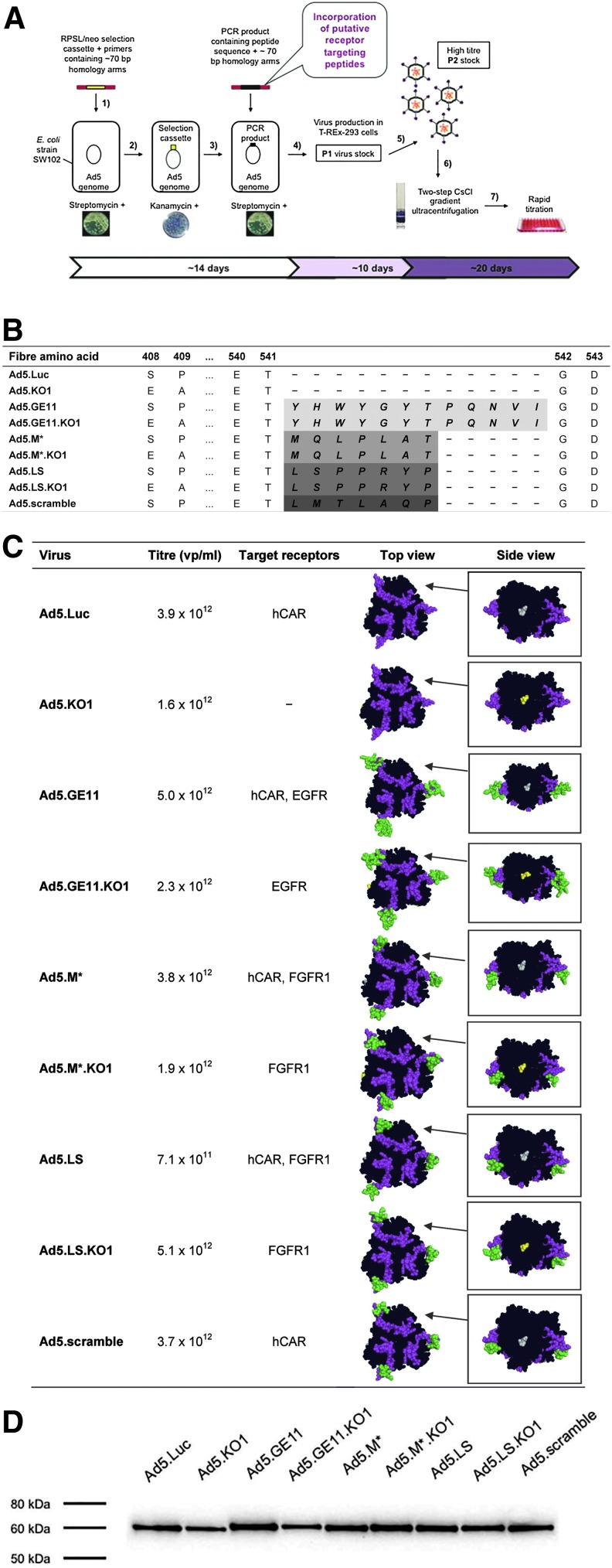
Generation of recombinant cancer-targetable Ad5 vectors. **(A)** Overview of viral modification and production using recombineering. (1) Design of the selection cassette, (2) temperature-induced (42°C) recombineering of the selection cassette into *Escherichia coli* strain SW102 by electroporation, (3) recombineering of the target sequence by electroporation, (4) verification of the correct clone by sequencing, purification of DNA by maxiprep, and generation of P1 virus stocks in permissive T-REx-293 cells, (5) propagation of high titer P2 stocks, (6) purification of viral particles by CsCl gradient ultracentrifugation and dialysis, and (7) titration by microBCA assay. **(B)** Amino acid sequence alterations within the Ad5 fiber knob domain. Peptide sequences *GE11*, YHWYGYTPQNVI; M*, MQLPLAT; *LS*, LSPPRYP; and *scramble*, LMTLAQP, were genetically inserted into fiber knob HI loop after Thr541. For the purpose of native hCAR-binding ablation, KO1 mutation (S408E, P409A) was introduced into the AB loop. **(C)** The inserted peptides (shown in green) *GE11*, M*, *LS*, *scramble*, and the HI loop (magenta) are highlighted. Inset: side view of the fiber knob, showing native (gray) or mutated hCAR-binding site (yellow). EGFR, epidermal growth factor receptor; FGFR1, fibroblast growth factor receptor 1; hCAR, coxsackie and adenovirus receptor; KO1, hCAR-binding mutation. **(D)** Verification of fiber integrity by Western blotting. Color images available online at www.liebertpub.com/hum

We generated a panel of genetically modified Ad5 vector genomes by rapid AdZ homologous recombineering method (Fig. 1A), based on red lambda genetics.^31^ DNA sequences encoding small peptide agonists of EGFR (36 bp) or FGFR1 (21 bp) were incorporated into the genome of replication-deficient (ΔE1/ΔE3) Ad5 vector genomes. The introduced modifications included insertion of GE11, M*, and LS peptides into the HI loop of Ad5 fiber knob domain after Thr541 (Fig. 1B and C) since this region has previously been described as permissive of incorporation of small peptide-based targeting ligands.^21,34^ A scrambled version of the M* peptide (Ad5.scramble) was also created to act as a negative control to ensure that the possible peptide-mediated virus entry is dependent on the specific peptide sequence. Detargeted Ad5 vectors were engineered by introduction of KO1 point mutations (S408E, P409A) into the peptide-modified re-targeted vectors (Fig. 1B and C) in an attempt to abrogate cell entry via hCAR. All modifications were confirmed by sequencing to ensure that no mutation had occurred within the region of homologous recombination. Viruses could be grown to high titers (Fig. 1C), indicating that peptide incorporation within the virion had no adverse effects on viral infectivity.

### Validation of fiber integrity following peptide insertion

The recombinant fiber monomers incorporating GE11, M*, LS, and scramble peptide insertions were assessed for their structural conformity by using the Ad5 fiber knob (Protein Data Bank ID: 1KNB) as a template for the 3D models (Fig. 1C). All 3D structures predicted the peptide insert to be presented in a conformation compatible with receptor interactions, extending outward from the fiber knob domain. All recombinant Ad5 vectors showed a clear and distinctive single band of ∼60 kDa on Western blotting (Fig. 1D), indicating that peptide insertion within the fiber HI loop had not affected the integrity of the resultant virion. The fiber monomer of modified vectors migrated slightly slower than Ad5.Luc, reflecting the increase in size due to peptide incorporation.

### Effect of peptide insertion on transduction *in vitro*

**FIG. 2.**
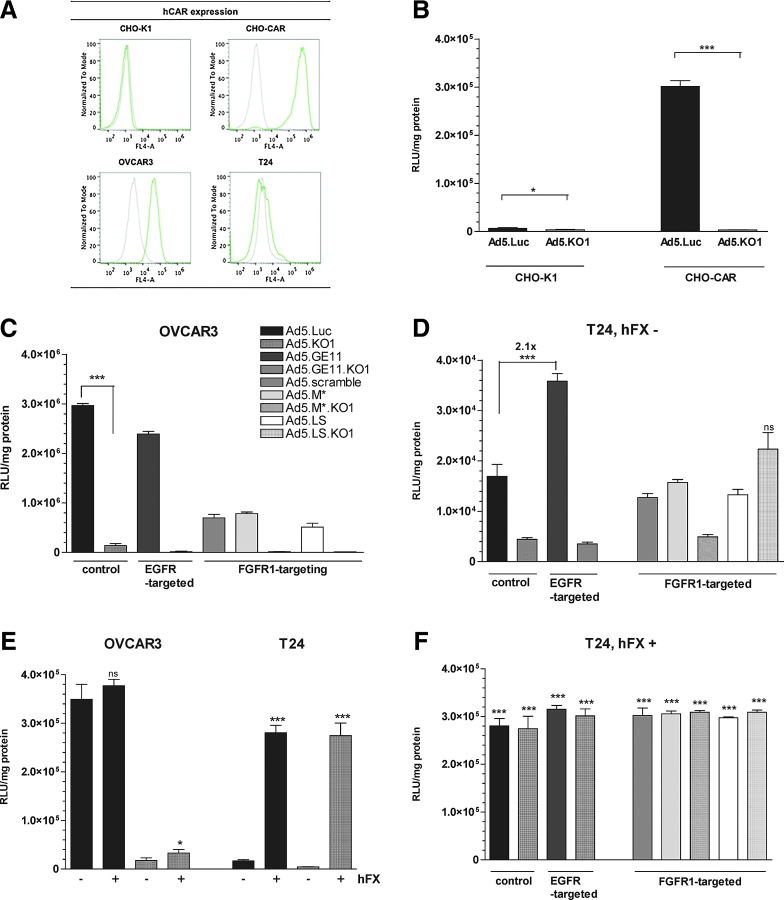
Transduction efficiency of targeted Ad5 vectors and effect of human coagulation factor X (hFX). **(A)** hCAR expression (green) as compared with isotype control normal mouse IgG (gray) on different cell lines, measured by flow cytometry. **(B)** Transduction efficiency on CHO-K1 and CHO-CAR cell lines in serum-free medium (*n*=4). **(C)** Transduction efficiency on OVCAR3 cells (*n*=3). **(D)** Transduction efficiency in serum-free medium (hFX^−^) on T24 cell line (*n*=4). **(E)** Transduction efficiency on OVCAR3 and T24 cell lines in serum-free medium or supplemented with 10 μg/ml of human coagulation factor X (hFX) (*n*=4). *p*-Values indicate comparison to the same virus in serum-free conditions (hFX^−^). **(F)** Transduction efficiency in presence of hFX (+) on T24 cell line (*n*=4). *p*-Values indicate comparison to the same virus in serum-free conditions (Fig. 2D). *p<0.05, **p<0.01, ***p<0.001, ns=not statistically significant, *p*>0.05. Error bars represent SD. Color images available online at www.liebertpub.com/hum

We evaluated the expression levels of hCAR, EGFR, and FGFR1 on a range of cell lines, as well as on primary patient-derived EOC cells, in order to select suitable model cell lines for evaluating adenovirus re-targeting efficacy. The cells were assessed for hCAR (Fig. 2A) and EGFR surface receptor expression by flow cytometry, while FGFR1 expression profiles were sourced from the literature (Table 1A). Four cell lines—CHO-K1, CHO-CAR, OVCAR3, and T24—were selected for further evaluation of transduction efficiency, based on their hCAR, EGFR, and FGFR1 expression profiles. Primary, patient-derived EOC cells showed high levels of hCAR and EGFR expression (Table 1B).

Table 1.Analysis of Cell Surface Receptor Expression by Flow CytometryA. Receptor Expression on a Panel of Human Cancer Cell Lines

**Table:** 

*Cell lines*		*Receptor expression*		
*Name*	*Origin*	*hCAR*	*EGFR*	*FGFR1*^a^
CHO-K1	Chinese hamster ovarian epithelial	−	−	−
CHO-CAR	CHO-K1 cell line expressing hCAR	+	−	−
OVCAR3	Human ovarian adenocarcinoma	+	+	+
T24	Human urinary bladder transitional cell carcinoma	−	+	+

^a^ FGFR1 expression on CHO-K1,^49^ OVCAR3,^27^ and T24^50^ cell lines.

**Table:** 

B. Receptor Expression on Primary Epithelial Ovarian Cancer (EOC) Cells from Ascitic Fluid, at Passage 2	

*Primary EOC cells*	
*Morphology*	*Receptor expression*
	

EGFR, epidermal growth factor receptor; EOC, epithelial ovarian cancer cells; FGFR1, fibroblast growth factor receptor; hCAR, human coxsackie and adenovirus receptor.

Cell morphology was monitored to exclude the possibility of fibroblast contamination.

An amount of 2.5×10^5^ cells were stained with primary antibodies mouse anti-hCAR (1:500, green), mouse anti-EGFR (1:200, blue), mouse anti-FGFR1 (1:100, red), and isotype control normal mouse IgG (1:200, gray) in triplicate and detected with secondary goat antimouse AlexaFluor647 conjugate (1:500). An amount of 2×10^4^ gated events were recorded in FL-4 on BD Accuri B6 flow cytometer. Color images available online at www.liebertpub.com/hum

To confirm that the KO1 mutations (Ser408Glu and Pro409Ala) could efficiently ablate hCAR-mediated cell infectivity, we used model CHO-K1 (hCAR^low^) and CHO-CAR (hCAR^high^) cell lines. As expected, Ad5 efficiently transduced CHO-CAR cells, but not CHO-K1 cells (Fig. 2B). Introduction of the KO1 abolished transduction of CHO-CAR cells (transduction levels similar to CHO-K1 cells), thus substantiating the involvement of these amino acids in hCAR engagement (Fig. 2B).

In OVCAR3 cells (hCAR^high^), levels of transduction for Ad5.GE11, Ad5.M*, and Ad5.LS were decreased compared with parental Ad5.Luc (Fig. 2C; *p*<0.001). Surprisingly, when the re-targeting peptides were presented in combination with hCAR ablation (KO1 vectors), we did not observe any increase in transduction compared with the parental Ad5.KO1 vector, implying a significant role for hCAR in uptake of the recombinant viruses in these cells. In T24 cells, which are hCAR^low^, incorporation of the GE11 peptide resulted in a significant (*p*<0.01) ∼2-fold increase in transduction compared with parental Ad5 (Fig. 2D), while no increase in transduction was evident for either of the FGFR1-targeted viruses, Ad5.M* or Ad5.LS (Fig. 2D). Since T24 cells are hCAR^low^, incorporation of the KO1 mutation did not significantly change transduction efficiency of the parental vector. However, we were surprised that when KO1 mutation was combined with the GE11, M*, or LS insertion, we failed to observe any detectable increases in levels of transduction in this hCAR^low^/EGFR^high^/FGF^high^ cell line.

### Effect of physiological concentrations of hFX on viral transduction levels

Interaction between the Ad5 hexon protein and the blood clotting factor, hFX, has been shown previously to underlie the hepatic tropism of intravenously administered Ad5, by bridging the virus to HSPGs on hepatocytes. In order to evaluate the effect, if any, of peptide incorporation in the fiber protein on the capacity of our panel of viruses to infect cancer cell lines in the presence of hFX, we performed transduction assays in the presence and absence of physiological hFX concentrations (10 μg/ml). In hCAR^high^ OVCAR3 cells, Ad5.Luc transduction was unaffected by hFX (Fig. 2E), with a modest ∼2-fold increase for Ad.KO1, while in hCAR^low^ T24 cells in which basal levels of transduction were low (Fig. 2E), hFX significantly enhanced transduction by 1–2 orders of magnitude, independent of peptide insertion (Fig. 2F). Similar observations were made for the control virus Ad5.KO1 on CHO-K1 and CHO-CAR cell lines (data not shown), as hFX was capable of rescuing transduction even in the case of fully abrogated hCAR-mediated entry pathway. Therefore, for the generated vectors to be useful for intravenous cancer therapeutics, additional mutations will be required with the hexon protein to preclude such interactions.

### Neutralization of vector transduction by ascitic fluid from ovarian cancer patients

**FIG. 3.**
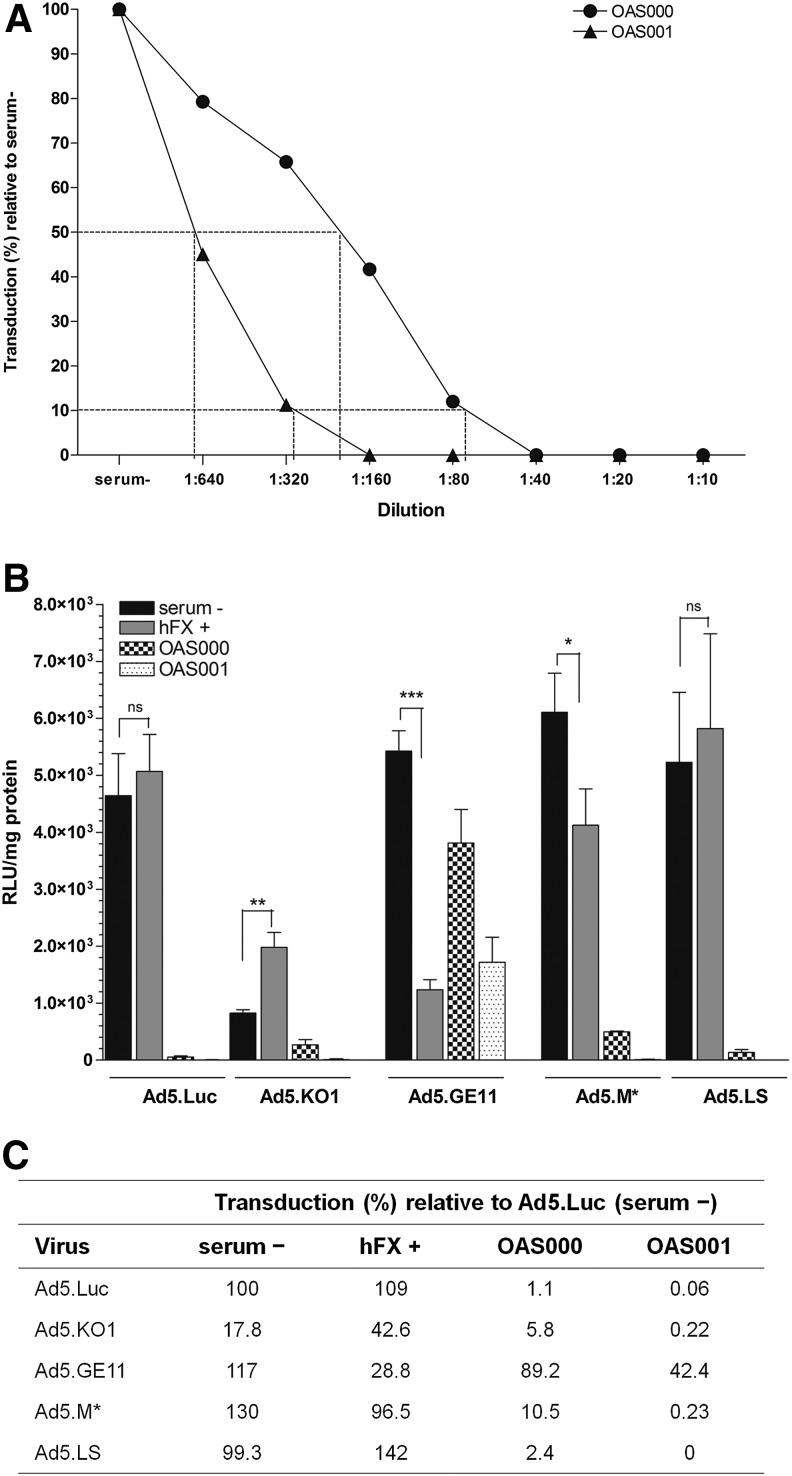
Transduction efficiency on primary epithelial ovarian cancer (EOC) cells and vector neutralization by ascitic fluid. **(A)** Inhibition of Ad5.Luc vector transduction in the presence of increasing concentrations of ascitic fluid, relative to serum^–^ conditions. The approximate dilution that neutralizes 50% and 90% of transduction is indicated with dotted lines. **(B)** Vector transduction efficiency in four different virus–medium preincubation conditions: serum^−^ medium; with 10 μg/ml of human coagulation factor X (hFX+); with 2.5% OAS000 supernatant; or with 2.5% OAS001 supernatant. **(C)** Neutralizing effect of ovarian ascites fluid supernatant on Ad5 vector transduction. Transduction levels (%) are shown relative to Ad5.Luc transduction in serum-free conditions. OAS000, ovarian ascites supernatant patient #000; OAS001, ovarian ascites supernatant patient #001. Error bars represent the SD (*n*=3).

To evaluate the potential of the generated vectors as agents for local delivery in ovarian cancer, we obtained ascitic fluid from patients with ovarian cancer, and established primary EOC cell cultures. We performed transduction assays in OAS001 EOC cells. First, to establish the neutralizing antibody titers in ascitic fluid, we performed a series of transduction experiments using Ad5.Luc in the presence of doubling dilutions of ascitic fluid. We found that Ad5-mediated transduction was inhibited by >90% by ∼1/320 or ∼1/80 dilution of OAS000 or OAS001, respectively, with 50% inhibition noted at dilutions of ∼1/640 and ∼1/160, respectively (Fig. 3A). Based on these findings and previous literature,^11^ we selected a neutralizing dose of 1/40 (2.5%) for further studies. Viruses were preincubated in serum^−^ medium or medium supplemented either with hFX or with 2.5% cell-free ascitic fluid obtained from patients OAS000 and OAS001 consistent with previous studies evaluating the nAb responses to Ad-based vectors from patient isolates.^11,35^

In the presence of hFX, only Ad5.KO1 demonstrated a significant increase in transduction efficiency (*p*=0.002), while hFX actually reduced transduction for Ad5.GE11 (*p*<0.001) and Ad5.M* (*p*=0.022) compared with transduction in the absence of serum or hFX (Fig. 3B and C). In the presence of 2.5% ascitic fluid, levels of transduction with Ad5 were reduced by 98.9% and 99.9% for OAS000 and OAS001, respectively (Fig. 3B and C), when compared with serum-free conditions, consistent with the presence of extremely high levels of preexisting nAbs in ascitic fluid from these patients.^36^ However, when Ad5.GE11 was utilized, we noted a significantly reduced pattern of neutralization, with transduction reduced to 89.2% and 42.4% of that in the negative control (Ad.Luc in serum-free conditions) for OAS000 and OAS001, respectively (Fig. 3B). Thus, the presence of the 12-mer peptide was able to augment transduction by 81- and 707-fold, respectively, in the presence of 2.5% neutralizing ascites, suggesting that peptide incorporation was able to facilitate viral escape from nAbs. A smaller, but significant response was also noted for the 7-mer M* peptide, with transduction enhanced by 10- and 4-fold in the presence of 2.5% neutralizing sera.

## Discussion

Achieving tumor-selective delivery of adenoviral anticancer vectors via the systemic route is highly problematic because of the myriad of “off-target” interactions with cells and proteins in the blood that dictate tropism and toxicity. Ultimately, these dose-limiting interactions limit bioavailability and efficacy. Ovarian cancer represents an ideal avenue for localized delivery of oncolytic virotherapies via the peritoneal route, circumventing some of the difficulties of delivery via the intravenous route.

In order to generate Ad vectors appropriate for localized therapeutic applications in ovarian cancer, we generated a suite of genetically modified vectors that combine mutations within the Ad5 fiber protein that ablate interactions with the native Ad5 receptor, hCAR, with peptide insertions which re-target the recombinant viral vectors to receptors widely recognized as being upregulated in tumors, namely, EGFR and FGFR1. The selected peptides were YHWYGYTPQNVI (GE11) binding to EGFR^22^ and two heptapeptides, MQLPLAT (M*)^23^ and LSPPRYP (LS),^24^ binding to FGFR1. To the best of our knowledge, none of these peptides have been tested previously for their ability to re-target a viral vector, though previous studies for GE11 have demonstrated the capacity of this peptide to target nanoparticles,^37^ drugs,^38^ nonviral gene therapy vectors,^39,40^ and chemically modified Ad vectors^41^ via the EGFR receptor, while the M* peptide has been utilized previously for targeted delivery of nonviral vectors.^23,42^

We demonstrate that although we were able to generate all the viral vectors to high titers, we were only able to observe modest re-targeting from hCAR to EGFR using Ad5.GE11 in the EGFR^high^CAR^low^ cell line, T24. We were unable to observe any obvious re-targeting from hCAR to FGFR1, using the Ad5.M*- or Ad5.LS-based vectors. Here, we have utilized peptides selected previously by screening linear bacteriophage libraries, for genetic re-targeting strategies within a constrained viral protein. It would appear that the constraints of the genetic approach imposing secondary structure on the targeting peptide may have reduced the affinity of the selected peptide for the target receptor, although previous strategies utilizing the same approach have yielded success.^43–47^

A key limitation to the clinical deployment of Ad-based medicines is the high level of preexisting immunity, estimated at >90% in some populations, which results in rapid sequestration, neutralization, and elimination of virotherapy, following systemic administration. The immunodominant epitopes remain a subject of debate, with a number of publications suggesting that Ad5 hexon hypervariable regions (HVRs) are the major site of neutralization, at least following intramuscular challenge. Indeed, several publications have elegantly demonstrated how genetically engineering the Ad5 HVRs to exchange them for those from rarely isolated serotypes results in viral vectors capable of escaping immune recognition in preimmunized mice and monkey models.^13^ However, following native respiratory infection with Ad5, the Ad fiber protein is overexpressed and secreted from epithelial cells to facilitate apical escape of the virus, where is interacts with hCAR on the basolateral membrane, disrupting the tight junction integrity and allowing Ad5 virions to emerge apically.^48^ Therefore, it is highly conceivable that a native Ad5 infection would result in a predominantly antifiber response, as opposed to a dominant antihexon response following intramuscular challenge with Ad5.

Here, we demonstrate that the supernatant derived from ovarian ascites at 2.5% readily neutralizes Ad5 infectivity^36^ by up to 3 logs. To our surprise, we also found that Ad5.GE11 was highly resistant to this neutralization under the same conditions, suggesting that the incorporation of the 12-mer peptide in the fiber knob domain impairs the capacity of host antibodies in ascetic fluid to bind and neutralize the Ad5-based vector. We noted a smaller but significant effect for the Ad5.M* virus in evading preexisting immunity. Ad5.M* displays a 7-mer FGFR1 interacting motif within the fiber knob protein, and it is unclear whether the smaller effect noted is because of the lack of expression of the FGFR1 receptor in the EOC cells tested (which were EGFR+/FGFR1−) or because of the smaller peptide motif having a less marked effect on masking immunodominant epitopes within the fiber knob domain.

Taken together, we propose that the peptide incorporation within the fiber protein represents a potentially powerful means for circumventing preexisting Ad5 immunity in clinical populations and could represent promising means for improving Ad5 efficacy clinically for local applications such as in ovarian cancer.

## Supplementary Material

Supplemental data
