# Supplementary material for: Incorporation of Peptides Targeting EGFR and FGFR1 into the Adenoviral Fiber Knob Domain and Their Evaluation as Targeted Cancer Therapies
Source: Hum Gene Ther. 2015 Apr 27;26(5):320–9. doi: 10.1089/hum.2015.015 (PMC4442602; doi:10.1089/hum.2015.015)
Supplement: Supplemental data [file Supp_Data.pdf]

Supplementary Data

Supplementary Table S1. Primer Sequences for Fiber Modifications

A. Primers Used to Generate DNA Fragments for Insertion Through Homologous Recombination

Primer	*Sequence (5′–3′)*
GE11 F	CTTAAACGGAGACAAAACTAAACCTGTAACACTAACCATTACACTAAACGGTACACAGGAAACATACCACTGGTACGGCTACACCCCCCAGAACGTGATC
GE11 R	TGTAGTTGTGGCCAGACCAGTCCCATGAAAATGACATAGAGTATGCACTTGGAGTTGTGTCTCCGATCACGTTCTGGGGGGTGTAGCCGTACCAGTGGTA
M* F	TGTCAGTCAAGTTTACTTAAACGGAGACAAAACTAAACCTGTAACACTAACCATTACACTAAACGGTACACAGGAAACAATGCAGCTGCCCCTGGCCACC
M* R	CAAATATTTCATTAATGTAGTTGTGGCCAGACCAGTCCCATGAAAATGACATAGAGTATGCACTTGGAGTTGTGTCTCCGGTGGCCAGGGGCAGCTGCAT
LS F	TGTCAGTCAAGTTTACTTAAACGGAGACAAAACTAAACCTGTAACACTAACCATTACACTAAACGGTACACAGGAAACACTGTCCCCCCCCCGGTACCCC
LS R	CAAATATTTCATTAATGTAGTTGTGGCCAGACCAGTCCCATGAAAATGACATAGAGTATGCACTTGGAGTTGTGTCTCCGGGGTACCGGGGGGGGGACAG
Scramble F	TGTCAGTCAAGTTTACTTAAACGGAGACAAAACTAAACCTGTAACACTAACCATTACACTAAACGGTACACAGGAAACACTGATGACCCTGGCCCAGCCC
Scramble R	ATGAGTTAGCTCCAAATGTTGCTCCGATATAGCTTCCGTTAGTCCAGGTGTATGAAAATGACATTGAGTATGCACTGGTGGCTGGGCCAGGGTCATCAG
RPSL-neo HI loop F	GTCAGTCAAGTTTACTTAAACGGAGACAAAACTAAACCTGTAACACTAACCATTACACTAAACGGTACACAGGAAACACCTGTGACGGAAGATCACTTCG
RPSL-neo HI loop R	CAAATATTTCATTAATGTAGTTGTGGCCAGACCAGTCCCATGAAAATGACATAGAGTATGCACTTGGAGTTGTGTCTCCCTGAGGTTCTTATGGCTCTTG
RPSL-neo AB loop F	AGTTTTGACAGCACAGGTGCCATTACAGTAGGAAACAAAAATAATGATAAGCTAACTTTGTGGACCACACCAGCTCCACCTGTGACGGAAGATCACTTCG
RPSL-neo AB loop R	CTGTAGCAAGTATTTGACTGCCACATTTTGTTAAGACCAAAGTGAGTTTAGCATCTTTCTCTGCATTTAGTCTACAGTTCTGAGGTTCTTATGGCTCTTG
KO1 mutation oligo	GAAACAAAAATAATGATAAGCTAACTTTGTGGACCACACCAGCTCCAGAGGCCAACTGTAGACTAAATGCAGAGAAAGATGCTAAACTCACTTTGGTCTT

Regions with homology to Ad5.Luc DNA sequence are underlined. F, forward primer; GE11, YHWYGYTPQNVI; KO1, hCAR-binding mutation; LS, LSPPRYP; M*, MQLPLAT; R, reverse primer; scramble, LMTLAQP.

B. Sequencing Primers

Primer	*Sequence (5′–3′)*
HI loop	GCCTATACAAACGCTGTTGG
AB loop	GTTCACCTAATGCACCAAACAC

Ad5 fiber knob HI loop was sequenced to verify successful peptide insertions and AB loop was sequenced to verify the KO1 (S408E, P409A) mutation.
